# Social-cognitive functioning and social skills in patients with early treated phenylketonuria: a PKU-COBESO study

**DOI:** 10.1007/s10545-016-9918-0

**Published:** 2016-02-25

**Authors:** Rianne Jahja, Francjan J. van Spronsen, Leo M. J. de Sonneville, Jaap J. van der Meere, Annet M. Bosch, Carla E. M. Hollak, M. Estela Rubio-Gozalbo, Martijn C. G. J. Brouwers, Floris C. Hofstede, Maaike C. de Vries, Mirian C. H. Janssen, Ans T. van der Ploeg, Janneke G. Langendonk, Stephan C. J. Huijbregts

**Affiliations:** 1 University of Groningen, University Medical Center Groningen, Beatrix Children’s Hospital, Division of Metabolic Diseases CA33, PO Box 30.001, 9700 RB Groningen, The Netherlands; 2 Department of Clinical Child and Adolescent Studies & Leiden Institute for Brain and Cognition, Leiden University, Leiden, The Netherlands; 3 Department of Developmental and Clinical Neuropsychology, University of Groningen, Groningen, The Netherlands; 4 Academic Medical Center, Amsterdam, The Netherlands; 5 Laboratory Genetics Metabolic Diseases, University Hospital Maastricht, Maastricht, The Netherlands; 6 Wilhelmina Children’s Hospital, University Medical Center Utrecht, Utrecht, The Netherlands; 7 University Medical Center St Radboud Nijmegen, Nijmegen, The Netherlands; 8 Center for Lysosomal and Metabolic Diseases, Erasmus Medical Center, Rotterdam, The Netherlands

## Abstract

**Objective:**

Early treatment of phenylketonuria (ET-PKU) prevents mental retardation, but many patients still show cognitive and mood problems. In this study, it was investigated whether ET-PKU-patients have specific phenylalanine (Phe-)related problems with respect to social-cognitive functioning and social skills.

**Methods:**

Ninety five PKU-patients (mean age 21.6 ± 10.2 years) and 95 healthy controls (mean age 19.6 ± 8.7 years) were compared on performance of computerized and paper-and-pencil tasks measuring social-cognitive abilities and on parent- and self-reported social skills, using multivariate analyses of variance, and controlling for general cognitive ability (IQ-estimate). Further comparisons were made between patients using tetrahydrobiopterin (BH4, *N* = 30) and patients not using BH4. Associations with Phe-levels on the day of testing, during childhood, during adolescence and throughout life were examined.

**Results:**

PKU-patients showed poorer social-cognitive functioning and reportedly had poorer social skills than controls (regardless of general cognitive abilities). Quality of social-cognitive functioning was negatively related to recent Phe-levels and Phe-levels between 8 and 12 years for adolescents with PKU. Quality of social skills was negatively related to lifetime phenylalanine levels in adult patients, and specifically to Phe-levels between 0 and 7, and between 8 and 12 years. There were no differences with respect to social outcome measures between the BH_4_ and non-BH_4_ groups.

**Conclusion:**

PKU-patients have Phe-related difficulties with social-cognitive functioning and social skills. Problems seem to be more evident among adolescents and adults with PKU. High Phe-levels during childhood and early adolescence seem to be of greater influence than current and recent Phe-levels for these patients.

## Introduction

In phenylketonuria (PKU; OMIM 212600), the brain dysfunction is, at least in part, considered to be the result of dopamine and serotonin deficiency (Blau et al 2010). Dopamine depletion has repeatedly been related to deficits in executive function (EF), which is the most frequently reported cognitive deficit in ET-PKU (Christ et al 2010), but also with impairments in social cognition (Skuse and Gallagher 2009). Decrease of serotonin activity has also been associated with (orbitofrontal cortex-mediated) EF-problems (Gellynck et al 2013), deficits in social cognition and social behaviour, and with mood disorders (Kiser et al 2012; Lesch and Waider 2012). Whereas a Phe-restricted diet with amino acid supplements is still the most widely-used treatment strategy for PKU, a sub-group of patients benefits (at least with respect to keeping Phe-levels in the lower ranges) from the use of tetrahydrobiopterin (BH_4_), which is a co-factor of the deficient enzyme phenylalanine hydroxylase (PAH). In many patients Phe-levels clearly rise after childhood, which, in turn, may affect dopamine and serotonin levels (MacDonald et al 2010; Trefz et al 2011). Higher Phe-levels are permitted in many national guidelines, and it is not yet fully clear what the upper target Phe-levels should be in adolescence and adulthood in relation to cognitive ability, social and behavioural functioning, necessitating more research in these age groups.

Social cognition involves all mental processes that underlie social interactions and comprises the ability to perceive, to interpret and to respond appropriately to social cues. Basic social-cognitive skills include face recognition, emotion recognition and Theory of Mind (ToM), i.e. the ability to attribute and understand thoughts, feelings and intentions to/of other people (Hughes and Leekam 2004). Although social cognition and social functioning have not yet been studied extensively in PKU, it seems likely that the consistently reported EF-problems (Christ et al 2010) extend to these domains, because of shared underlying neurobiology and neuro-anatomy (Arnsten and Rubia 2012; Heinz et al 2011; Murphy et al 2002). Also, several mental health problems, characterized by social (-cognitive) impairments, such as mood swings and depression, have been observed in PKU (Anjema et al 2011; Jahja et al 2013; Ten Hoedt et al 2011). In PKU a lack of autonomy and a low rate of forming normal adult relationships have been observed, although among early- and well-treated patients, such problems seem quite rare (Bosch et al 2007; Simon et al 2008).

The goal of the present study was to investigate social cognition and social skills of PKU-patients in relation to metabolic control, i.e. historical and concurrent Phe. As adherence is increasingly difficult with age, and because social demands increase from adolescence onwards, we hypothesized that problems would become more serious with increasing age, being the most severe in adults with PKU.

It was further expected that, in concordance with well-documented Phe-EF/IQ associations (Huijbregts et al 2002; Waisbren et al 2007), problems with social cognition and social skills would be related to both historical and concurrent Phe, with somewhat stronger associations for historical Phe-levels.

## Method

### Participants

The PKU group consisted of 95 patients (41 males, 54 females, mean age 21.6 years ± 10.2, range 7.0–42.8 years). All patients were born in or after 1974, when neonatal screening was introduced in The Netherlands, and were treated since diagnosis early after birth. All patients paid regular follow-up visits to one of six Dutch university medical centres (and were treated according to National Instituted of Health (NIH) guidelines, published in 2000). The healthy matched control group included 95 subjects (30 males, 65 females, mean age 19.6 years ± 8.7, range 7.2–40.8 years). Seven controls were siblings or cousins of the patients. The PKU and control groups did not differ significantly in age and gender distribution (Table 1). No differences were observed regarding SES-related measures (e.g. mean family income or achieved education levels) either. Although in the normal range, the PKU group had a lower IQ (101 ± 12) than controls (108 ± 13): *t*(186) = 3.8, *p* < 0.001.

**Table 1:** Descriptive statistics for PKU and control group, per age group

	PKU < 12 years	Controls < 12 years	PKU 12–17 years	Controls 12–17 years	PKU ≥ 18 years	Controls ≥ 18 years
	(*n* = 26)	(*n* = 27)	(*n* = 13)	(*n* = 15)	(*n* = 56)	(*n* = 53)
Gender (male:female)	11:15	12:15	5:8	4:11	25:31	14:39
Mean age ± SD (range)	9.3 ± 1.6 (7.0–12.0)	9.3 ± 1.4 (7.2–11.6)	15.3 ± 1.7 (12.8–17.5)	15.1 ± 1.6 (12.8–17.9)	28.9 ± 6.5 (18.7–42.8)	26.0 ± 5.8 (18.1–40.8)
Mean concurrent Phe ± SD (range)	350 ± 202 (153–944)		531 ± 311 (130–1250)		657 ± 336 (66–1550)	
Lifetime Phe ± SD (range)	297 ± 74 (199–490)		326 ± 125 (212–707)		467 ± 156 (230–882)^a^	
Lifetime Phe 0–7 years ± SD (range)	279 ± 72 (158–445)		257 ± 96 (185–522)		340 ± 158 (175–1048)^d^	
Lifetime Phe 8–12 years ± SD (range)	336 ± 93 (207–612)^b^		309 ± 134 (194–717)		371 ± 178 (165–1130)^e^	
Lifetime Phe 13–17 years ± SD (range)			509 ± 188 (303–914)^c^		505 ± 240 (222–1079)^e^	
Lifetime Phe >18 years ± SD (range)					617 ± 251 (226–1173)^e^	

^a^ *n* = 53. Three incomplete lifetime Phe

^b^ *n* = 21

^c^ *n* = 11

^d^ *n* = 52

^e^ *n* = 53

Blood samples were taken from the patients preprandially at home on the day of neuropsychological testing, which took place at the patients’ clinics. Historical Phe-levels were collected from hospital databases. Lifetime Phe was calculated as the mean of half-year median Phe-levels, from birth until the day of testing. Also, Phe-levels between 0–7, 8–12, 13–17 and ≥18 years were calculated when applicable, to gain more insight into Phe-levels during particular developmental stages. PKU-patients had a mean concurrent Phe-level of 556 μmol/L ± 328. Mean lifetime Phe was 399 μmol/L ± 154 (*n* = 92; lifetime Phe of three patients was for the larger part incomplete and could therefore not be calculated) (Table 1). Concurrent and lifetime Phe were correlated with each other (*r* = 0.67, *p* < 0.001). Age was significantly related to concurrent (*r* = 0.37, *p* < 0.001) and lifetime Phe (*r* = 0.55, *p* < 0.001), indicating higher Phe-levels for older patients. Historical Phe-levels between 0 and 7 years were higher for adults with PKU than for children and adolescents with PKU (*F*(2,88) = 3.2, *p* = 0.046).

Thirty patients used tetrahydrobiopterin (BH_4_-) doses up to 20 mg/kg with a maximum of 1400 mg/day. The BH_4_ group was younger (mean age 15.8 ± 8.5) than the non-BH_4_ group (mean age 24.4 ± 9.9): *t*(65) = 4.4, *p* < 0.001. Lifetime (*t*(90) = 6.3, *p* < 0.001) and concurrent Phe (*t*(90) = 5.0, *p* < 0.001) were lower for the BH_4_ group.

### Instruments

Children performed three tasks to measure social-cognitive abilities; adolescents and adults performed four tasks. In order to assess social skills, for children and adolescents the Social Skills Rating System (SSRS; Gresham and Elliot 1990) was filled out by parents, whereas adults filled out the Social Skills Checklist-Self Report (SSC-SR; Novotni 2000). Subtests Vocabulary and Block Design from the Wechsler Intelligence Scale for Children (WISC-III, Wechsler 1991) or the Wechsler Adult Intelligence Scale (WAIS-III, Wechsler 1997) were used to estimate verbal and performance IQ.

Two tasks were performed by all participants: the computerized Face Recognition and (FR) and Identification of Facial Emotions (IFE) tasks from the Amsterdam Neuropsychological Tasks (ANT; De Sonneville 2014). FR examines how fast and how well participants are able to recognize neutral faces and consists of three parts (40 trials each): faces were presented from the front, ‘en profile’, and upside down. Each trial is preceded by a target face, after which participants have to decide whether the subsequent signal, containing four faces, includes the target face. In the IFE, participants identify facial emotions as quickly and accurately as possible. The task has four parts (40 trials each), where consecutively happy, sad, angry and frightened faces have to be identified. The mean of age-corrected z-scores for accuracy and RT on the FR and IFE was calculated, and converted so that a higher score indicated a better performance, leading to one overall z-score representing social cognition as measured by computerized tasks.

One task was performed only by children up to the age of 12. This paper-and-pencil (P&P-) task, the Social Cognitive Skills Test (Van Manen et al 2007), examines ToM-skills. The task consists of seven stories with associated pictures. After each story, questions were asked representing eight aspects of ToM, leading to a total score with four levels. The standardized total score was used as dependent variable. A high score indicates better ToM. Two tasks were performed only by adolescents and adults. These P&P-tasks, the Faux-Pas Recognition Test (FPT; Baron-Cohen et al 1999) and the Reading the Mind in the Eyes (RME-) task (Baron-Cohen et al 2001) measure ToM. FPT measures how one interprets social situations that are potentially awkward (‘faux pas’). It consists of five stories containing a faux pas and four control stories, followed by questions about the social occurrence in the story. A higher score indicates a better ability to interpret social situations. This score was again standardized for statistical analyses. RME measures how well mental states of others are understood based solely on information from the eyes. The test consists of 36 photographs of eye regions of individuals, of which participants have to identify the best fitting emotion from four possible answers. A higher (standardized) score indicates a better ability to recognize emotions. Mean z-score for accuracy of the three P&P-tasks was used in statistical analyses.

Regarding the questionnaires, the SSRS consists of 38 items, measuring social skills on four different dimensions (cooperation, assertiveness, responsibility and self-control), thereby using 3-point rating scales. Higher scores represent better social skills. The SSC consists of 81 items, measuring social skills on eight different dimensions (basic manners, nonverbal and verbal communication, communication roadblocks, organizational skills, self-control, knowledge, relationships and self-care), using 3-point rating scales. Higher scores indicate better social skills. Standardized mean score for the SSRS and SSC-R was used to represent the level of social skilfulness.

### Data analyses

As noted, standardized scores, calculated separately for the computerized tasks, P&P-tasks and questionnaires were used in statistical analyses (performed with IBM SPSS Statistics 22^nd^ version). For all measurements, higher z-scores indicated better performance on the social cognition tasks or better social skills.

General linear model (GLM-) multivariate analyses of variance were conducted for all participants, then separately for participants <12 years, 12–17 years and ≥18 years. Within the separate age-group analyses further statistical control for age was exerted (by including it as a covariate) when applicable, as within each age group developmental influences may still be present. Social cognition-computerized, social cognition-P&P and social skills (all z-scores) were used as dependent variables. Analyses were repeated with IQ as a covariate. Comparisons between BH_4_-treated and non-BH_4_-treated patients were also made using t-tests. Associations between social outcomes and lifetime/concurrent Phe were investigated using Pearson’s correlations.

## Results

Table 2 shows scores on tasks and questionnaires. Performance on the computerized tasks was related to performance on the P&P (*r* = 0.48, *p* < 0.001). There were no significant associations between task-performance and scores on the questionnaires, indicating they measure different constructs, i.e. social cognition versus social skills. Higher IQ was significantly correlated with performance on the computerized tasks (*r* = 0.35, *p* < 0.001) and P&P (*r* = 0.51, *p* < 0.001).

**Table 2:** Descriptive statistics of all social measurements for PKU and control group, per age group

		PKU < 12 years	Controls < 12 years	PKU 12–17 years	Controls 12–17 years	PKU ≥ 18 years	Controls ≥ 18 years
		(*n* = 26)	(*n* = 27)	(*n* = 13)	(*n* = 15)	(*n* = 56)	(*n* = 53)
ANT							
	FR, % errors	17.0 ± 8.0	15.0 ± 8.4	9.2 ± 4.5	6.8 ± 4.4	7.1 ± 3.5	5.6 ± 3.4
	FR, reaction time	1965 ± 493	1716 ± 454	1221 ± 324	1129 ± 174	1197 ± 272	1133 ± 221
	IFE, % errors	10.7 ± 6.2	9.6 ± 6.8	8.6 ± 4.9	6.3 ± 2.5	5.7 ± 3.1	4.6 ± 2.5
	IFE, reaction time	1304 ± 276	1082 ± 263	844 ± 113	807 ± 103	874 ± 182	796 ± 157
P&P							
	SCST	130.1 ± 23.7	138.3 ± 13.5				
	RME			22.4 ± 3.4	23.2 ± 2.9	24.4 ± 3.9	26.1 ± 3.6
	FPT			49.6 ± 5.0	54.6 ± 2.8	49.2 ± 5.1	51.3 ± 4.6
Questionnaires							
	SSRS	59.6 ± 11.1	61.3 ± 9.8	60.6 ± 12.3	67.5 ± 7.2		
	SSC					140.8 ± 17.2	149.9 ± 8.6

*ANT* Amsterdam neuropsychological tasks, *P&P* paper-and-pencil tasks, *Q* questionnaires, *FR* face recognition, *IFE* identification of facial emotions, *SCST* social cognitive skills task, *RME* reading the mind in the eyes, *FPT* faux-pas recognition test, *SSRS* social skills rating system, *SSC* social skills checklist

Note 1. Values are given as mean absolute score and standard deviation

Note 2. P&P and Q: higher score indicates better outcome

### MANOVAs PKU versus controls

#### All ages

**Fig. 1:**
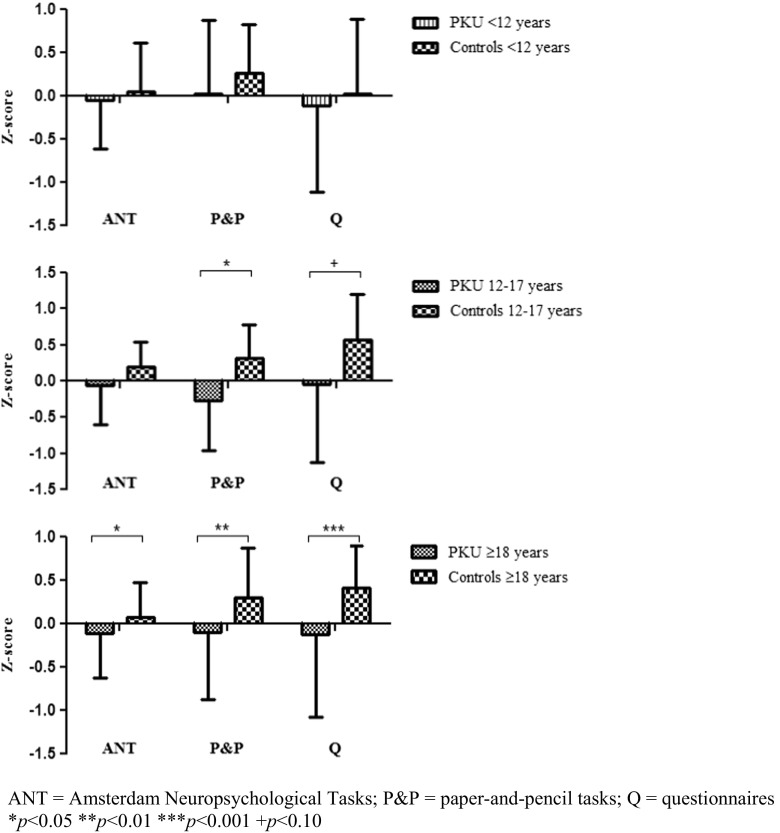
Outcome per age group on social measures for PKU and control group (higher z-scores indicate better outcomes). *ANT* Amsterdam neuropsychological tasks, *P&P* paper-and-pencil tasks, *Q* questionnaires. *p < 0.05 **p < 0.01 ***p < 0.001 + *p* < 0.10

The multivariate effect showed that there was a significant difference between patients and controls: *F*(3185) = 9.70, *p* < 0.001, *η* ^*2*^_*p*_ = 0.14. On all measurements, controls outperformed or scored better than PKU-patients (Fig. 1, Table 3). When age was included as a second independent variable (<12 versus 12–17 versus ≥18 years), the group effect remained significant: *F*(3181) = 7.58, *p* < 0.001, *η* ^*2*^_*p*_ = 0.11. No significant interaction effects between age and group were found, indicating that the multivariate group differences were the result of differences between patients and controls in all age groups. After including IQ as a covariate, significant group differences remained for social cognition as measured by P&P-tests and social skills. A comparison between BH_4_ and non-BH_4_ users, controlling for age, did not show significant differences. As the lack of group by age interactions may have been due to decreasing statistical power by adding a variable with three levels (<12 versus 12-17 versus ≥18 years) or to the fact that cut-offs for developmental stages simply do not lie exactly at 12 and 18 years, we were interested in the results for separate age groups. Repeating the analyses for the separate age groups resulted in the following:

**Table 3:** Statistical overview MANOVAs for PKU versus controls

		*F*	*df*	*η* ^*2*^ *p*
All ages				
	ANT	6.07*	1187	0.031
	P&P	14.95***	1187	0.074
	Q	12.92***	1187	0.065
Age <12 years				
	ANT	0.38	1,50	0.008
	P&P	1.40	1,50	0.027
	Q	0.29	1,50	0.006
Age 12–17 years				
	ANT	2.39	1,26	0.084
	P&P	7.19*	1,26	0.217
	Q	3.42+	1,26	0.116
Age ≥18 years				
	ANT	4.42*	1107	0.040
	P&P	9.12**	1107	0.079
	Q	13.22***	1107	0.110

*ANT* Amsterdam neuropsychological tasks, *P&P* paper-and-pencil tasks, *Q* questionnaires

*p < 0.05 **p < 0.01 ***p < 0.001 + *p* < 0.10

#### Children, age <12 years

There were no differences regarding the multivariate effect or any of the univariate effects (Table 3), indicating PKU-patients and controls under the age of 12 scored equally well on the social cognition-tasks and on the questionnaire assessing social skills. When including age or IQ as a covariate, results remained similar.

#### Adolescents, age 12–17 years

The multivariate effect for adolescents was *F*(3,24) = 6.33, *p* = 0.003, *η* ^*2*^_*p*_ = 0.44. There was a significant difference between the adolescent PKU and control groups on P&P and a trend for social skills (Table 3), which was irrespective of age and IQ.

#### Adults, age ≥18 years

The multivariate effect was *F*(3105) = 6.82, *p* < 0.001, *η* ^*2*^_*p*_ = 0.16. PKU adults performed worse than controls on all social measures (Table 3). However, when including age as covariate, only the differences regarding P&P and social skills remained significant. When IQ was taken into account, there was only a significant group difference for social skills and a trend for P&P. Nonetheless, the multivariate group effect remained significant after including age and IQ.

### Associations between metabolic control and social outcomes

#### All ages

Higher lifetime Phe was significantly related to poorer social skills (*r* = −0.19, *p* = 0.036). Concurrent Phe was not significantly related to any of the outcome measures. Controlling for age or IQ did not alter these associations. Within the BH_4_ group, the relationship between lifetime Phe and social skills was not significant. Examining lifetime Phe in more detail per age group (<12, 13–17, ≥18 years) gave the following results:

#### Children, age <12 years

No significant associations between social measurements and lifetime and concurrent Phe were observed.

#### Adolescents, age 12–17 years

Significant associations between P&P and Phe between 8 and 12 years (*r* = −0.52, *p* = 0.033), and between 13 and 17 years (*r* = −0.51, *p* = 0.055) were present. There were no significant associations with concurrent Phe.

#### Adults, age ≥18 years

Lifetime Phe and social skills were significantly related (*r* = −0.26, *p* = 0.031). Specifically, social skilfulness was correlated with Phe between 0 and 7 years (*r* = −0.28, *p* = 0.022), between 8 and 12 years (*r* = −0.24, *p* = 0.041) and (almost significantly) between 13 and 17 years (*r* = −0.21, *p* = 0.064). Partial correlations with age as a covariate showed similar results. No significant associations with concurrent Phe were observed.

## Discussion

Adolescents and adults with PKU show impaired social-cognitive functioning. Adult patients also report difficulties with social skills compared to controls. Higher lifetime rather than concurrent Phe-levels were related to poorer social outcomes. Strength of effects varied, with larger effect sizes for group differences between adolescent and adult patients and their healthy counterparts (cf. Cohen, 1988, 1994). The strength of correlations between Phe-levels during different age-periods and social and social-cognitive outcomes also varied with moderate-to-large associations for Phe between 0 and 12 years and social-cognitive functioning of adolescent patients and social skills of adult patients. Despite the fact that it remains difficult to judge the importance of these statistically meaningful results, evidence is provided that there are Phe-related problems with social-cognitive abilities and social skills in PKU, which, considering their importance for several other functional domains and everyday life, should therefore feature in treatment and monitoring.

Phe between 0 and 7 years was higher for the adult patients than for the PKU children and adolescents, possibly indicating changes in treatment regimen throughout the years. As our results showed that low Phe during childhood and early adolescence is important for social functioning later in life, this might indicate good news for the current generation of children (and adolescents) with PKU. PKU children in the present study generally had Phe-levels below the recommended upper target levels (360 μmol/L) and there were no differences with healthy controls and no associations between Phe-levels and social outcomes for this age group. Mean lifetime Phe-levels were below the upper target limit for adolescents (600 μmol/L) as well, but they had poorer social-cognitive abilities than their healthy counterparts. These findings could be interpreted as evidence for recommending even lower upper target levels throughout childhood or at least adolescence. However, social (-cognitive) functioning of PKU-patients may be determined by many other factors in addition to Phe-levels. Considering the additional burden (which might even work counterproductively when social functioning is concerned) of an even stricter treatment regimen, it would be premature to interpret these results as evidence supporting the lowering of upper target Phe-levels.

Patients using BH_4_ had lower lifetime and concurrent Phe, probably partly because BH_4_-responsive patients generally present with milder forms of PKU, and partly because of the treatment itself. There were no differences in social outcomes between the BH_4_ and non-BH_4_ groups. Also, no associations between Phe and social measures in the BH_4_ group were found. Thus, inclusion of patients using BH_4_ may have resulted in fewer-than-expected associations between Phe and social outcomes. It is also possible that lowering Phe-levels through BH_4_ simply does not result in better social-cognitive abilities and social skills, or only does so in the longer term.

The present study also showed that deficits in social skills were not fully explained by impaired general cognitive ability. The possibility still exists that EF is a larger contributor to social skills than IQ. As EF-problems are the most prominent cognitive deficits observed in PKU, future studies should examine associations between EF and social outcomes in more detail. Furthermore, factors such as family functioning, upbringing by parents (e.g. being overprotected), dietary compliance and contents, level of stress experienced either through coping with PKU or through other sources (e.g. parental divorce) should be taken into account when investigating social functioning in PKU (MacDonald et al 2010; Olsson et al 2007).

The fact that, for this study, we did not take into account many other factors, apart from Phe-levels, that might be related to social outcomes may be considered a limitation. Another issue that needs to be taken into account is that all patients were recruited through treatment centres, which implies that, at least, they were followed up (regardless of how well they adhered to their treatment regimens). Patients lost to follow-up may very well show higher Phe-levels (as there is no external monitoring in place at all) and subsequent poorer social (-cognitive) functioning. Thus, in reality, when all patients are considered, group differences and associations between social (-cognitive) functioning and Phe could be more pronounced. It may also be considered a limitation that we used parent- and self-reports to measure social skills. Although it is very difficult to assess social functioning in laboratory settings (which generally do not mimic daily life situations very well), it is possible that reports partly depended on social metacognition. However, in this respect it is noteworthy that group differences regarding social skills remained significant after statistical control for general cognitive ability. A further issue that warrants attention in future studies is that differential prediction from Phe-levels during different age periods is complicated by the fact that they are generally related, which could lead to multicollinearity. Therefore, in future studies it would be preferable if different statistical approaches could be taken, that distinguish developmental patterns or trajectories of Phe-levels.

The present study demonstrates deficits in core social (-cognitive) abilities. The influence of Phe during childhood and early adolescence seems to be important for social-cognitive functioning and social skills later in life. Overall, this study showed the importance of assessment of social (-cognitive) functioning in PKU-patients.

## Abbreviations

ANT: Amsterdam Neuropsychological Tasks
EF: Executive function(ing)
FPT: Faux-Pas Recognition Test
FR: Face Recognition
IFE: Identification of Facial Emotions
Phe: Phenylalanine
PKU: Phenylketonuria
PKU-COBESO: Phenylketonuria COgnition, BEhavior, SOcial functioning study
RME: Reading the Mind in the Eyes Test
SCST: Social Cognitive Skills Test
SSC-SR: Social Skills Checklist-Self Report
SSRS: Social Skills Rating System
Tyr: Tyrosine
WISC-III: Wechsler Intelligence Scale for Children 3^rd^ Edition
WAIS-III: Wechsler Adult Intelligence Scale 3^rd^ Edition
